# Android Robot-Mediated Mock Job Interview Sessions for Young Adults with Autism Spectrum Disorder: A Pilot Study

**DOI:** 10.3389/fpsyt.2017.00169

**Published:** 2017-09-11

**Authors:** Hirokazu Kumazaki, Zachary Warren, Blythe A. Corbett, Yuichiro Yoshikawa, Yoshio Matsumoto, Haruhiro Higashida, Teruko Yuhi, Takashi Ikeda, Hiroshi Ishiguro, Mitsuru Kikuchi

**Affiliations:** ^1^Department of Clinical Research on Social Recognition and Memory, Research Center for Child Mental Development, Kanazawa University, Ishikawa, Japan; ^2^Departments of Pediatrics and Psychiatry, Vanderbilt University, Nashville, TN, United States; ^3^Department of Psychiatry and Behavioral Sciences, Vanderbilt University, Nashville, TN, United States; ^4^Department of Systems Innovation, Graduate School of Engineering Science, Osaka University, Osaka, Japan; ^5^JST ERATO ISHIGURO Symbiotic Human-Robot Interaction, Osaka, Japan; ^6^Service Robotics Research Group, Intelligent Systems Institute, National Institute of Advanced Industrial Science and Technology, Ibaraki, Japan

**Keywords:** autism spectrum disorder, android robot, robotic intervention, job interview, vocational training

## Abstract

The feasibility and preliminary efficacy of an android robot-mediated mock job interview training in terms of both bolstering self-confidence and reducing biological levels of stress in comparison to a psycho-educational approach human interview was assessed in a randomized study. Young adults (ages 18–25 years) with autism spectrum disorder (ASD) were randomized to participate either in a mock job interview training with our android robot system (*n* = 7) or a self-paced review of materials about job-interviewing skills (*n* = 8). Baseline and outcome measurements of self-reported performance/efficacy and salivary cortisol were obtained after a mock job interview with a human interviewer. After training sessions, individuals with ASD participating in the android robot-mediated sessions reported marginally improved self-confidence and demonstrated significantly lower levels of salivary cortisol as compared to the control condition. These results provide preliminary support for the feasibility and efficacy of android robot-mediated learning.

## Introduction

An estimated 50,000 individuals with autism spectrum disorder (ASD) turn 18 each year and begin the process of transitioning to adult-based services in the United States. Unfortunately, the employment rate for adults with ASD is quite low (1, 2), highlighting the need for supports, programs, and tools that can assist with aspects of acquiring and keeping jobs. A number of programs have recently started to focus on establishing pathways toward employment and have highlighted the value of specific transition programs (3). Such programs have noted that a substantial initial barrier toward competitive employment can be the job interview itself (4, 5). There is evidence that greater self-confidence in one’s ability to perform a job interview is associated with greater social engagement during interviews as well as more effective verbal and non-verbal communicative strategies during interviews (6, 7). In this capacity, programs that improve both perceived and objective interview performance may be a critical component of addressing challenges regarding social communication and interaction for the ASD population that interfere with job acquisition.

Recent work has demonstrated the preliminary feasibility and efficacy of virtual reality job interview training with adults reporting potential improvements in self-confidence and demonstrating improved performance in mock interviews (8, 9). Recent advances in such virtual reality interventions may extend to the development of robotic interventions. In this capacity creating intelligent three-dimensional learning environments wherein individuals interact with robotic systems may represent another, potentially more powerful, avenue for enhancing skills with generalization to real-world settings (10).

In an effort to help facilitate job interview skill training for young adults with ASD, we developed a mock job interview training using an android robot, which is a robot with the appearance and movements resembling those of an actual human. Previous studies have reported use of an android robot system to support medical and welfare fields by means of communication (11). An advantage of using an android robot for mock job interview, compared to screen-based approaches, is that individuals can be exposed to a three-dimensional learning experience that more closely resembles the challenging and potentially anxiety-provoking situation of job-interviewing in a controlled manner.

In the current work, we examined feasibility of android robot-mediated interview training in a group of young adults with ASD by measuring self-report regarding self-confidence/performance and salivary cortisol levels. Greater self-confidence is important in tackling the target situation (12). Salivary cortisol provides a reliable, non-invasive metric of stress (13) and has been used to measure response to social situations (14–16). There is also evidence that cortisol is associated with self-confidence (17). In addition, recent work has suggested the importance of measuring both the physiological arousal level and the self-reporting score (18, 19) in individuals with ASD, which had already been utilized in a previous robot intervention study for typically developed subjects (20, 21). Therefore, we sought to assess both the self-reporting and physiological measures of arousal, to obtain a more objective view of the self-confidence in individuals with ASD.

## Materials and Methods

### Participants

Participants were young adults with ASD recruited from a Japanese medical center with specialization in treating developmental disorders. All procedures involving human participants were conducted in accordance with the ethical standards of the institutional and/or national research committee and with the 1964 Helsinki Declaration and its later amendments or comparable ethical standards. After a complete explanation of the study, all the participants provided written, informed consent. All participants agreed to participate in the study. Inclusion criteria included: (1) confirmed diagnosis of ASD based on DSM-5 criteria from supervising study psychiatrist (22), (2) ages 18–25 years, (3) unemployed workers who were actively seeking employment, and (4) scores over 30 on the Liebowitz Social Anxiety Scale (LSAS) (23) to confirm the presence of social anxiety. This clinician-administered scale consists of 24 items, 13 describing performance situations and 11 describing social interaction situations. Each of the items is separately rated for “fear” and “avoidance” using a 4-point categorical scale. Receiver operating curve analyses have shown that an LSAS score of 30 is correlated with minimal symptoms and is the best cutoff point for distinguishing between individuals with and those without social anxiety disorder (24, 25). This specific anxiety criterion was utilized for this preliminary study with the assumption that primary mechanism of effect of the android robot-mediated intervention would be related to reduced anxiety rather than skill enhancement *per se* (i.e., exposure tasks were relatively short series of content limited interactions with system).

Exclusion criteria included identified genetic and medical conditions (e.g., FMR1, Rett syndrome) from a registry. Child and adolescent psychiatrists collected information from guardians concerning developmental milestones (including joint attention, social interaction, pretend play, and repetitive behaviors, with onset prior to 3 years of age) and episodes (e.g., how the individual with ASD behaved at kindergarten and school). Additional professionals, such as teachers and social workers, provided further background based on their detailed observations of interactions with people (particularly non-family members), repetitive behaviors, obsessive/compulsive traits, and stereotyped behaviors. The first author confirmed the existing diagnoses using both diagnostic instruments and screening questionnaires, including the Pervasive Developmental Disorder–Autism Society Japan Rating Scale (PARS), which is a diagnostic interview-based scale for ASD developed in Japan (26). Sub and total scores of this scale correlate with the domain and total scores of the Autism Diagnostic Interview-Revised (27, 28).

All participants completed the Autism Spectrum Quotient-Japanese version (AQ-J) (29), which was used in the evaluation of ASD-specific behaviors and symptoms. The AQ-J is a short questionnaire with five subscales (social skills, attention switching, attention to detail, imagination, and communication). Previous work with the AQ-J have been replicated across cultures (30) and age (31, 32). The AQ is sensitive to the broader autism phenotype (33). IQ was measured by either the Wechsler Intelligence Scale for Children—Fourth Edition or the Wechsler Adult Intelligence Scale—Third Edition.

### Procedures

**Figure 1:**
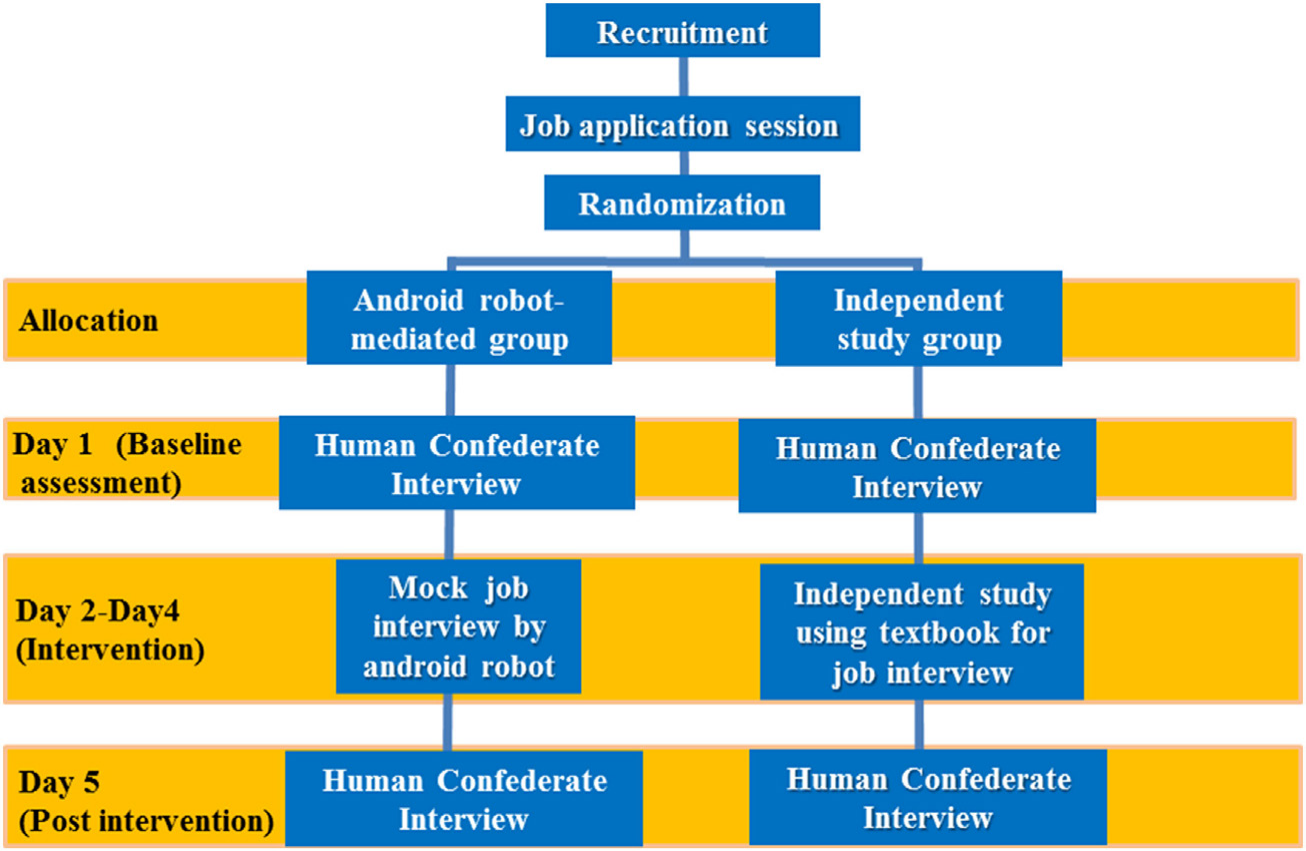
Participant flow.

Initially, participants completed a mock job application in which they chose from six potential jobs to which to apply, with questions concerning this job carried into the mock job interview sessions. Then, individuals were randomly assigned to two groups (see Figure 1): android robot-mediated mock interviewing or independent study, subsequent to this application. Over five consecutive days, the subsequent trial procedures were conducted from day 1 to day 5. Each participant always began the experiment (mock job interview with a human interviewer or android robot, or independent study) at the same time of day. On days 1 and 5, participants of both groups participated in a 10-min mock job interview with a human interviewer. During this mock job interview, the human interviewer followed a specific interview script and protocol across all interviews. From day 2 to day 4, participants in the android robot-mediated group participated in a similar 10-min mock job interview, for three consecutive days. Across sessions, the scripts were varied slightly to promote engagement, but followed the same basic structure. Please refer to Data Sheet in Supplementary Material for examples of the scripts.

**Figure 2:**
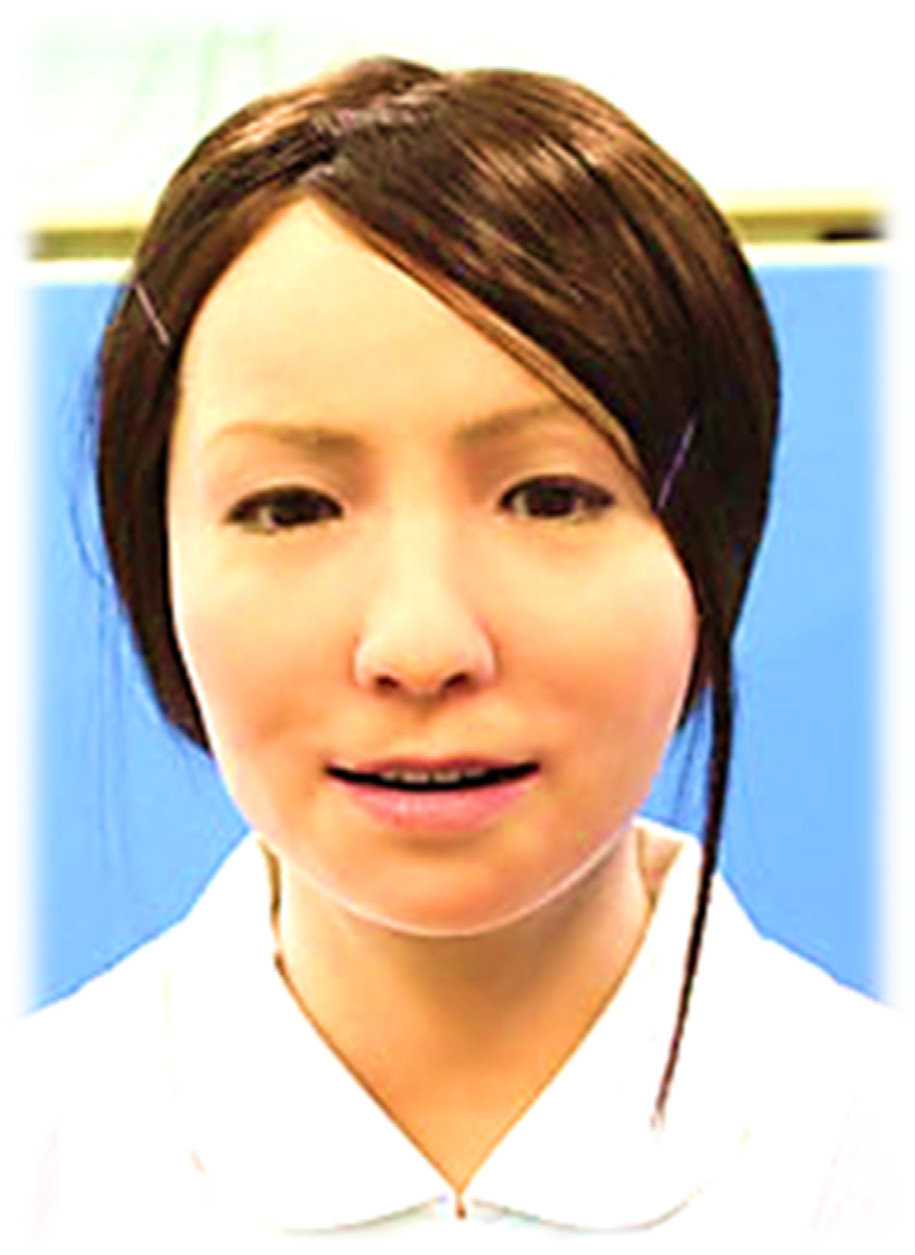
ACTROID-F (android robot).

**Figure 3:**
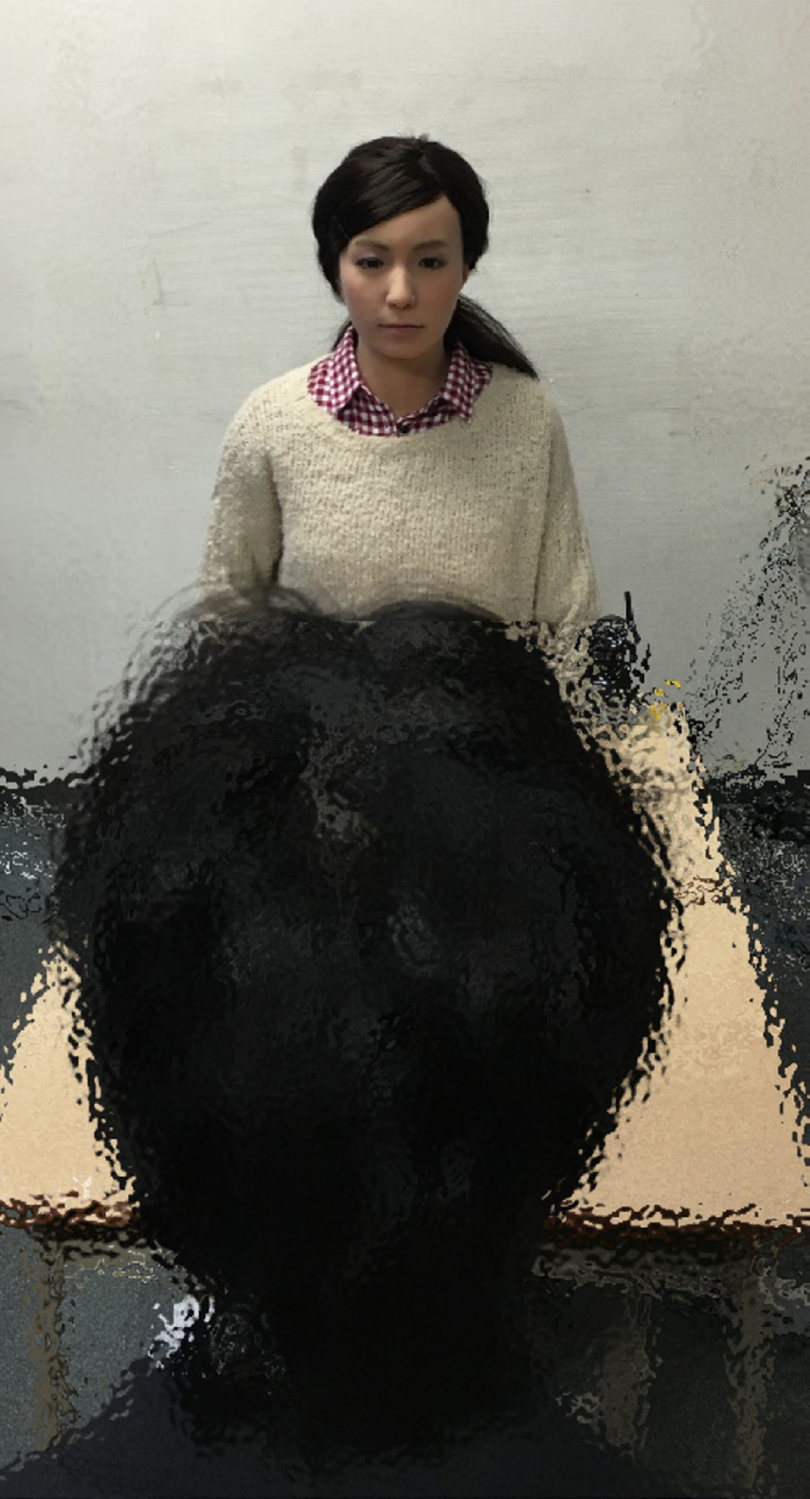
Example of how participants typically interacted with mock job interview by ACTROID-F.

The android robot used in this study was Actroid-F (Figures 2 and 3) (Kokoro Co. Ltd.), a female type of humanoid robot with an appearance similar to that of a real person (11). Its artificial body has the same proportions, facial features, hair color, and hairstyle as a human. To elicit the belief that the robots were behaving and responding autonomously, we adopted a remote control system similar to those conventionally used in robotics research (34). The Actroid-F was incorporated changes in facial expression (smiling, nodding, brow movements) during speech. Participants in the independent study group were encouraged to read and answer materials about question collection for which was often asked in real job interview daily for a minimum of 10 min (the approximate duration of the android robot-mediated sessions).

**Figure 4:**
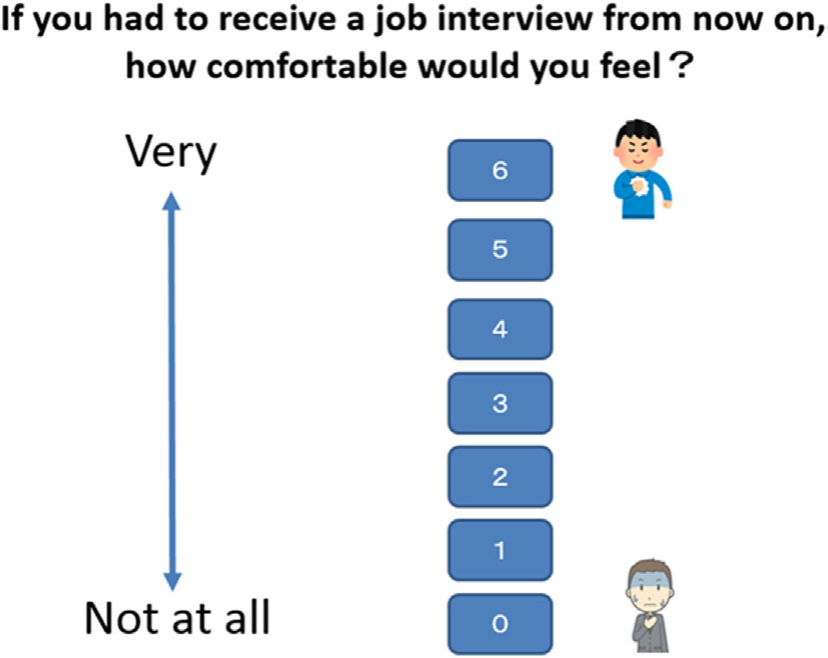
Confidence rating scale for receiving job interview.

After the human interviewer sessions, and at the same time each day (i.e., after experimental session, or at the scheduled time for the control condition), all participants were given questionnaires scored on Likert rating scales of self-confidence in performance. Ratings were from 0 (not at all comfortable) to 6 (very comfortable) (see Figure 4).

In addition, to evaluate physiological responses, participants also provided salivary cortisol after the human interviewer sessions and at the same time each day (i.e., after the experimental session or at a scheduled time for the control condition). Samples were obtained at the same time each day in each group to account for potential diurnal variation, 20 min post interview for the experimental group to account for time delays in activation, with participants asked not to eat in the hour prior to the interview. Saliva samples (0.5–2.0 ml) were collected directly into sterile plastic tubes (15 ml) by passive drool and immediately frozen by dry ice. The samples were stored at −80°C until assayed. After thawing at room temperature, the saliva samples were centrifuged at 4°C 1,500 × *g* for 10 min to remove large precipitants. Determination of saliva cortisol was performed using a cortisol enzyme immunoassay kit (Salimetrics, State College, PA, USA). Samples (25 µl) were treated according to the manufacturer’s instructions. Measurements were performed in duplicate. The optical density of the samples and standards were measured at wavelengths of 450 nm by a microplate reader (Bio-Rad, Richmond, CA, USA). Sample concentrations were calculated by MatLab-7 according to the relevant standard curve (35).

All procedures involving human participants were conducted in accordance with the ethical standards of the institutional and/or national research committee and with the 1964 Helsinki Declaration and its later amendments or comparable ethical standards.

### Statistical Analysis

We performed statistical analysis using SPSS version 24.0 (IBM, Armonk, NY, USA). Descriptive statistics for the sample were used. Salivary cortisol measurements were positive and skewed toward large values; thus, log transformation was performed to achieve approximate normality, and the transformed values were used for all cortisol analyses. The differences of age, IQ, AQ-J, and LSAS score between the groups were analyzed using an independent samples *t*-test. The difference in gender proportion was analyzed using the χ^2^-test. The overall study design involved two independent variables: group (android robot-mediated group vs. independent study group); and time [1: pre-intervention (Day 1), 2: Day 2, 3: Day 3,4: Day 4, 5: postintervention (Day 5)]. There were two dependent variables: salivary cortisol level and self-confidence rate. To investigate the time course difference between the two groups (i.e., android robot-mediated group vs. independent study group), a two-way ANOVA was used to analyze the collected data (salivary cortisol level or self-confidence rate) with one repeated factor (time) and one group factor. As a complimentary analysis, to ascertain whether it is adaptive for individual with ASD to do mock job interview using android robot, we compared salivary cortisol level between Day 1 and Day 2 using a paired *t*-test. We employed an alpha level of 0.05 for this complementary analysis.

## Results

### Feasibility and Participation

In total, 15 individuals with ASD took part in the study (see Table 1 for participant details). All participants completed the trial procedures without technological challenges or noted participant distress that would lead to session termination. We carefully observed participant performance and confirmed that all participants were concentrating during the trials and highly motivated from the start to finish of the experiment.

**Table 1:** Descriptive characteristics of android robot-mediated group and independent study group.

Characteristics	Android robot-mediated group (*n* = 7) (M, SD)	Independent study group (*n* = 8) (M, SD)	Statistics		
			*t* or χ^2^	*df*	*p*-Value
Age in years	23.1 (2.0)	23.4 (3.5)	*t* = −0.154	13	0.880
Gender (males:female)	6:1	6:2	χ^2^ = 0.268	1	0.605
Full scale IQ	75.3 (13.8)	69.9 (9.9)	*t* = 0.862	13	0.394
AQ-J	27.1 (1.9)	28.4 (3.2)	*t* = −0.882	13	0.394
LSAS	68.1 (19.1)	63.9 (22.3)	*t* = 0.394	13	0.700

*AQ-J, autism spectrum quotient, Japanese version; LSAS, liebowitz Social Anxiety Scale*.

*In the AQ-J, higher scores reflect a greater number of autism spectrum disorder-specific behaviors*.

*Parentheses indicate SD*.

### Preliminary Analyses

There were no significant differences between groups with regards to mean age (*p* = 0.88), gender proportion (*p* = 0.61), average IQ score (*p* = 0.39), total AQ-J score (*p* = 0.39), or total LSAS score (*p* = 0.70). We also examined descriptive statistics for salivary cortisol measurements.

### Primary Analyses

**Figure 5:**
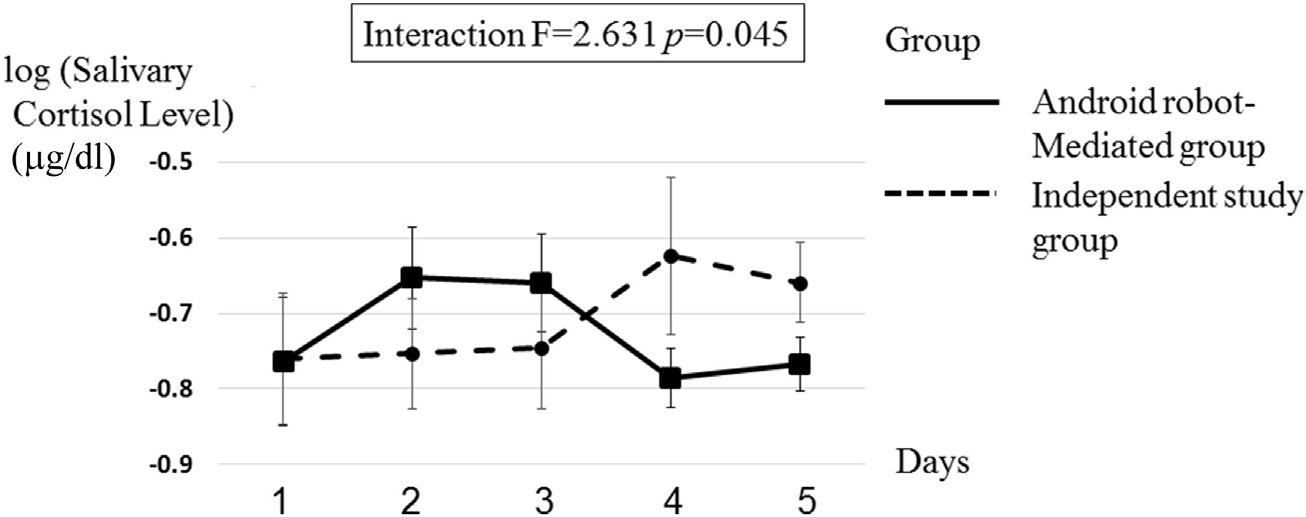
The mean salivary cortisol level in the android robot-mediated group, and independent study group.

**Figure 6:**
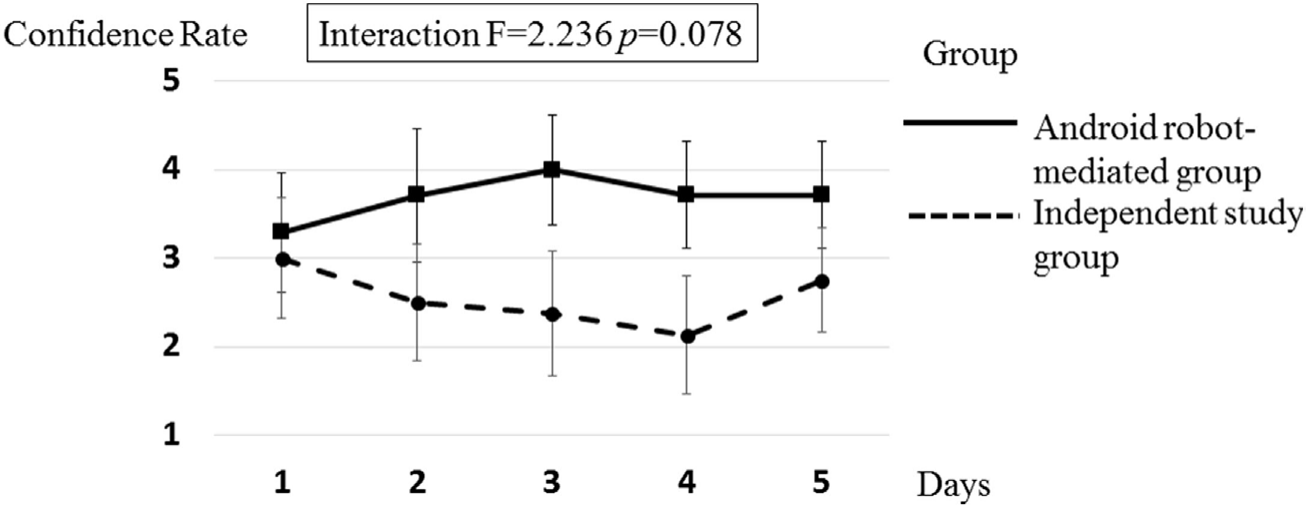
The mean confidence rate in the android robot-mediated group, and independent study group.

**Figure 7:**
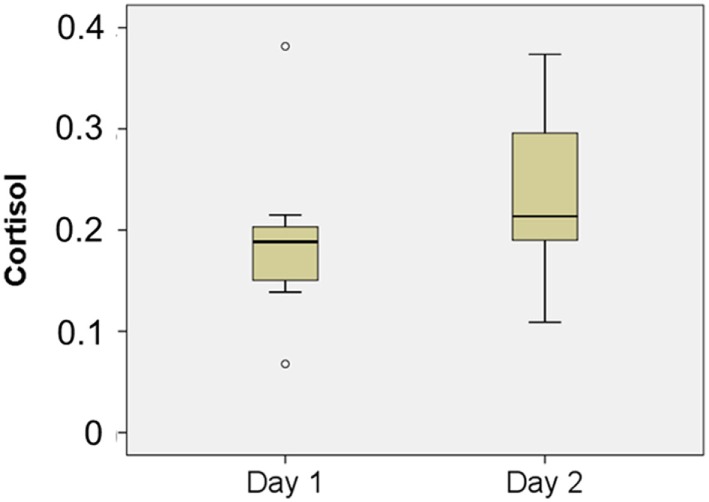
Comparison between log cortisol from Day 1 and Day 2 in android robot-mediated group. *p* = 0.04.

The overall study design involved two independent variables: group (android robot-mediated group vs. independent study group); and time [1: pre-intervention (Day 1) 2: post-intervention (Day 5)]. To investigate the time course difference between the two groups (i.e., android robot group vs. independent study group), two-way repeated measures ANOVAs were used to analyze the collected data on the primary outcomes of salivary cortisol levels and self-confidence ratings (see Table 2; Figures 5 and 6). Regarding salivary cortisol level, the two-way ANOVA resulted in significant interaction effect between group and time (*F* = 2.631; *p* = 0.045). Regarding ratings of self-confidence, the two-way ANOVA resulted in an interaction effect between group and time that approached significance (*F* = 2.236; *p* = 0.078). Given overall interaction effect, an examination of daily salivary cortisol change indicated a significant rise of salivary cortisol on Day 2 compared to Day 1 (*p* = 0.04) suggesting initial enhanced physiological arousal in the android robot-mediated interview setting (see Figure 7), with additional day-by-day analyses (2 to 3, 3 to 4, and 4 to 5) not revealing statistically significant changes.

**Table 2:** Means and SEM in android robot-mediated group and independent study group for confidence rating scale and salivary cortisol outcomes at baseline (day 1) and at post-intervention (day 5), and interaction effects between android robot-mediated group and independent study group for confidence rating scale and salivary cortisol outcomes.

Outcome	Group	Baseline (M, SEM)	Post-intervention (M, SEM)	*t* (Cohen’s *d*)	Statistics	
					*F* (η*p*^2^)	*p-*Value
Confidence rating scale	Android mediated	3.29 (0.68)	3.71 (0.61)	−1.162 (0.25)		0.289
	Independent study	3.13 (0.47)	3.20 (0.43)	−0.292 (0.14)		0.774
Interaction effect					2.236 (0.15)	0.078
log (Salivary cortisol level)	Android mediated	−0.76 (0.09)	−0.77 (0.04)	0.045 (0.02)		0.965
	Independent study	−0.76 (0.09)	−0.66 (0.05)	−1.525 (0.50)		0.171
Interaction effect					2.631 (0.17)	0.045*

*The effect size of difference between the baseline and post-intervention in confidence rating scale and salivary cortisol level was calculated by Cohen’s d. The effect size of differences between the baseline and post-intervention in the interaction effect of confidence rating scale and salivary cortisol level was calculated using partial eta squared*.

*p < 0.05.

## Discussion

In the current study, we studied the development and application of an android robot-medicated mock job interview training. The feasibility results suggest that participants tolerated exposure to the android robot-mediated sessions without issue (i.e., 100% completion) and were engaged enough to report alterations in performance over time relative to a simple self-study condition.

Significant rise of salivary cortisol between Day 1 and Day 2 in android robot-mediated group suggest enhanced physiological arousal in the new setting of mock job interview by android robot. It is initially easy to assume that elevated cortisol is deleterious and reflects a maladaptive stress response (36). However, it may be argued that under some conditions higher cortisol may be adaptive, and may be assumed that a moderate level of arousal is necessary for optimal performance for individuals with ASD (36, 37). Perhaps in individuals with ASD, a higher level of arousal or stress may be initially necessary to engage socially with novel peers (36). It is possible that the same is true of mock job interview by android robot in this study, considering the result that young adults with ASD who received job interview by android robot improved their self-confidence.

Young adults with ASD are able to self-report psychiatric symptoms, including anxiety (38). They may be more accurate reporters regarding their own mood dysregulation than their caregivers (37). Taken together, the results of the self-reporting questionnaire for self-confidence, which showed a trend toward improvement (although statistically marginally significant, because of the low statistical power of this pilot study) in self-confidence, supported the objective improvement in self-confidence in young adults with ASD.

The results of this preliminary efficacy study demonstrated that simple exposure to the android robot-mediated interview procedures contributed to the trend of self-reported increases in confidence, and importantly, to corresponding reductions in biological indicators of stress/anxiety. Interestingly, this occurred in absence of specific interview skill training (i.e., improvement just based on practice and exposure). Thus, the study provides preliminary evidence that utilizing robot-mediated systems, such as an android robot, may be acceptable/feasible and contribute to tangible improvements in functioning with potential for generalization to real-world settings. This finding of android robot-mediated role-playing increasing self-confidence is consistent with a number of recent studies suggesting changes in virtual reality and screen-based approaches (5, 8). Moreover, results of the current study suggest that three-dimensional learning environments wherein virtual agents are humanoid or android robot agents may also contribute to not only changes in self-reported performance but also seem to evoke levels of stress/anxiety that may be helpful to address in order to generalize performance to real-world settings.

The current study includes several areas of significant limitation. First, the group studied was small, with average cognitive skills, and was pre-selected for a base-level of anxiety symptoms. In addition, improvement of self-confidence was marginally significant (i.e., did not reach the statistical threshold). Clearly future work with a broader range of functioning individuals would be necessary for richer understanding of potential use and impact. Second, the comparison group participated in control self-directed learning condition and as such it is not possible to determine whether exposure to simple human-based mock interviewing, which is obviously much less resource intensive than an android robot-mediated protocol, would be just as efficacious. Third, while human interviewer interactions were utilized to understand outcomes relative to baseline, no specific performance criteria of interview skills were included, which limits our ability to comment on potential improvement beyond effects that are likely attributable to practice and exposure. Finally, the time frame of intervention and study was very short and not tied to specific vocational outcomes beyond the intervention setting, as such powerful questions about the impact of such a system remain.

Despite limits, this preliminary investigation represents a novel investigation into the feasibility of robot-mediated ASD intervention applications for young adults. While robotic technologies have been hypothesized as potential vehicles for enhancing skills in individuals with ASD, very few studies have tested such impact within experimental designs relevant to core areas of challenge or meaningful quality of life indicators. The current work provides preliminary support for a unique application of a robotic system (e.g., android robot-mediated interview exposure) to overcome a component (e.g., job interviewing) of a very specific challenge (e.g., employment) that many young adults with ASD struggle with over time. Future work of intelligent learning systems and robotic technologies may further leverage specific benefits of embodied interaction (e.g., attraction/appeal, perceived agency, diminished social challenge) to target and improve real-world skills for young adults with ASD in meaningful ways.

## Ethics Statement

The present study was approved by the ethics committee of the Kanazawa University. All procedures involving human participants were conducted in accordance with the ethical standards of the institutional and/or national research committee and with the 1964 Helsinki Declaration and its later amendments or comparable ethical standards.

## Author Contributions

HK designed the study, conducted the experiment, carried out the statistical analyses, analyzed and interpreted data, and drafted the manuscript. ZW, BC, YY, YM, HH, TI, HI, MK, and TY conceived of the study and participated in its design and assisted with data collection and scoring of behavioral measures and analyzed and interpreted the data and were involved in drafting the manuscript and revised it critically for important intellectual content. MK was involved in giving final approval of the version to be published. All authors read and approved the final manuscript.

## Conflict of Interest Statement

The authors declare that the research was conducted in the absence of any commercial or financial relationships that could be construed as a potential conflict of interest.
